# Development of Digital Teaching Materials on Coastal Ecology Based on Education for Sustainable Development to Enhance Geographic Literacy Among Senior High School Students in Coastal Areas

**DOI:** 10.12688/f1000research.184677.1

**Published:** 2026-06-30

**Authors:** Muhammad Ikhsan, Epon Ningrum, Mamat Ruhimat, Iwan Setiawan, Sony Nugratama Hijrawad, Irwan Abdullah, Salman Alparis Sormin, Tuti Purwati, Dwi Nanda Akhmad Romadhon

**Affiliations:** 1 Universitas Pendidikan Indonesia, Bandung, West Java, Indonesia

**Keywords:** coastal ecology education; digital teaching materials; Education for Sustainable Development; geographic literacy; ADDIE model; secondary education

## Abstract

**Background:**

Coastal ecosystems are among the most productive and socioeconomically important environments globally. However, students in coastal communities often exhibit limited geographic literacy regarding the ecological processes that sustain these ecosystems. This challenge is compounded by the limited availability of contextualized and sustainability-oriented digital learning resources.

**Objective:**

This study aimed to develop, validate, and evaluate the effectiveness of Education for Sustainable Development (ESD)-integrated digital teaching materials (DTMs) on coastal ecology in improving geographic literacy among senior high school students in coastal areas.

**Method:**

The study employed a Research and Development (R&D) approach using the ADDIE model, consisting of Analysis, Design, Development, Implementation, and Evaluation stages. Participants included 148 Grade XI students from four coastal-area schools selected through purposive sampling. The developed DTMs were validated by four subject-matter experts and three instructional technology specialists. Geographic literacy was assessed using a validated 40-item instrument covering spatial thinking, environmental knowledge, geospatial skills, and sustainability orientation. Effectiveness was examined through a quasi-experimental pre-test–post-test control group design.

**Results:**

The DTMs demonstrated high validity, with mean scores of 3.82/4.00 for content and 3.76/4.00 for media quality. Students in the experimental group showed a significant improvement in geographic literacy following the intervention (d = 0.84, p < .001), while the control group exhibited no significant change (p = .21). Among the assessed dimensions, sustainability orientation recorded the strongest effect (d = 0.91).

**Conclusion:**

ESD-integrated DTMs on coastal ecology effectively enhance geographic literacy among coastal secondary school students. The findings provide an empirically validated framework for integrating sustainability principles into maritime and geography education, particularly within developing archipelagic contexts.

## 1. Introduction

Coastal ecosystems — encompassing mangroves, coral reefs, seagrass beds, and estuaries — support approximately 40% of the global population through food provisioning, climate regulation, and livelihood generation ( Millennium Ecosystem Assessment, 2005; Spalding et al., 2017). In archipelagic nations such as Indonesia, where more than 17,000 islands generate over 95,000 km of coastline, the ecological literacy of coastal communities is not merely an academic concern but a foundational competency for sustainable resource governance ( Cicin-Sain et al., 2021; Rahmawati & Djahir, 2022). Despite this urgency, students residing in coastal areas frequently demonstrate limited understanding of the ecological and geographic processes governing these environments — a paradox that points toward structural deficiencies in contextually responsive geography education ( Lim et al., 2020; Syamsuddin et al., 2023).

Geographic literacy — defined as the capacity to think spatially, interpret environmental systems, and apply geospatial reasoning to real-world sustainability challenges — has emerged as a critical competency in twenty-first century education ( National Geographic Society, 2012; Bednarz et al., 2013; Solem et al., 2020). However, empirical studies consistently document its underdevelopment among secondary school students in coastal and maritime regions of the Global South, where geography curricula tend to privilege abstract content over place-based ecological reasoning ( Artvinli, 2020; Utami et al., 2022; Nugraha et al., 2023). This gap is compounded by the persistent absence of digital teaching materials (DTMs) that situate coastal ecology within frameworks of environmental responsibility and sustainability literacy.

Education for Sustainable Development (ESD), as articulated through UNESCO’s ESD for 2030 framework and enshrined in Sustainable Development Goal 4.7, offers a transformative pedagogical response to this challenge ( UNESCO, 2020). ESD positions learners not as passive recipients of environmental knowledge but as critical agents capable of understanding the systemic interdependencies between human activity and ecological sustainability. When operationalized through digital learning environments, ESD can integrate interactive simulations, place-based inquiry tasks, and reflective problem-solving in ways that traditional textbooks cannot achieve ( Michail et al., 2007; Wals, 2012; Brundiers et al., 2021). Nevertheless, the systematic integration of ESD principles into DTMs for coastal ecology at the senior secondary level remains largely unexplored in the empirical literature.

Existing research on geographic literacy interventions has predominantly focused on higher education settings ( Johansson, 2020; Kopnina, 2020) or has examined ESD broadly without disaggregating coastal ecology as a domain ( UNESCO, 2021). Studies addressing digital learning materials in Indonesian geography education tend to evaluate general usability and engagement rather than measuring literacy outcomes with validated instruments ( Pratama et al., 2022; Widoyoko et al., 2023). Critically, no published R&D study has developed and empirically evaluated ESD-integrated DTMs specifically targeting coastal ecology geographic literacy in secondary students from coastal communities — a gap that constrains evidence-based curriculum development in maritime education contexts.

To address these convergent gaps, this study developed, validated, and evaluated ESD-integrated digital teaching materials on coastal ecology (hereafter ESD-CoastalDTM) using the ADDIE instructional design model. Three research questions guided the inquiry: (1) To what extent do the developed DTMs meet expert validity criteria for content accuracy and media quality? (2) What is the effect of ESD-CoastalDTM on geographic literacy outcomes among coastal senior high school students? (3) Which dimensions of geographic literacy demonstrate the greatest responsiveness to the ESD-integrated intervention? By answering these questions, this study contributes: theoretically, by extending ESD theory to the domain of coastal geography education and specifying a testable literacy-development mechanism; methodologically, by demonstrating a validated geographic literacy instrument applicable across coastal school contexts; and practically, by producing a replicable DTM development framework for maritime-geography education in archipelagic developing nations.

## 2. Theoretical framework and literature review

### 2.1 Education for sustainable development as a pedagogical framework

ESD constitutes more than a content domain — it is a transformative approach to teaching and learning that cultivates the competencies necessary for individuals to contribute to a more sustainable world ( UNESCO, 2017; Rieckmann, 2018). The theoretical architecture of ESD rests on three interdependent pillars: normative orientation (values and ethics), cognitive engagement (critical and systemic thinking), and transformative action (agency and participation) ( Wals, 2012; Leicht et al., 2018). When translated into instructional design, these pillars require learning materials that move beyond declarative knowledge toward higher-order reasoning about ecological complexity, social justice, and intergenerational responsibility. Research in ESD-integrated curricula consistently demonstrates gains in environmental knowledge, pro-sustainability attitudes, and behavioral intention, though effect sizes vary substantially across contexts and age groups ( Boeve-de Pauw et al., 2015; Osman et al., 2021). Crucially, however, the empirical literature has yet to establish how ESD integration specifically affects domain-specific geographic literacy — the conceptual gap this study addresses.

### 2.2 Geographic literacy: Dimensions and measurement

Geographic literacy is a multidimensional construct encompassing spatial thinking, environmental knowledge, geospatial skill application, and sustainability orientation ( Bednarz et al., 2013; Solem et al., 2020; Massey, 2021). Spatial thinking involves reasoning about location, distribution, and spatial pattern; environmental knowledge encompasses understanding of ecological processes and human-environment interactions; geospatial skills refer to the ability to interpret maps, satellite imagery, and geographic information systems; and sustainability orientation reflects the disposition to apply geographic understanding toward equitable, long-term resource stewardship. While these dimensions are theoretically distinct, they are empirically interdependent: development in one dimension typically facilitates advancement in others ( Jo & Bednarz, 2009; Golledge et al., 2008). Research from coastal and maritime education contexts suggests that place-based instructional approaches — particularly those linking local ecological phenomena to broader systemic processes — are especially effective in developing all four dimensions simultaneously ( Ballantyne & Packer, 2009; Kudryavtsev et al., 2012; Utami et al., 2022).

### 2.3 Digital teaching materials in geography education

Digital teaching materials encompass any structured, technology-mediated instructional resource designed to achieve defined learning objectives — including e-modules, interactive multimedia, simulation environments, and augmented reality applications ( Hannafin & Land, 2000; Churchill, 2011). In geography education, DTMs have demonstrated particular promise for developing spatial visualization competencies, motivating inquiry into environmental processes, and bridging the gap between classroom learning and real-world geographic phenomena ( Kerski, 2013; Patterson et al., 2019). However, a systematic review by Pratama et al. (2022) found that DTMs in Indonesian secondary geography education rarely embed ESD principles explicitly, and fewer still have been developed for students in ecologically significant coastal areas. Furthermore, most existing DTMs are evaluated for user satisfaction and technical functionality rather than learning outcomes — a methodological limitation that constrains their evidence base for informing curriculum policy.

### 2.4 The ADDIE model as a development framework

The ADDIE (Analysis, Design, Development, Implementation, Evaluation) model provides a systematic, iterative framework for instructional product development that is widely adopted in educational technology research ( Branch, 2009; Peterson, 2003). Its structured phases enable rigorous alignment between learner needs assessment, learning objectives specification, material design, expert validation, field testing, and outcome evaluation — making it particularly suitable for R&D studies that seek both product validity and effectiveness evidence. Several recent studies applying ADDIE to geography DTM development in Indonesia have reported strong validity outcomes but have not assessed literacy-level impacts ( Widoyoko et al., 2023; Nugraha et al., 2023), reinforcing the need for the present study’s extended evaluation phase.

## 3. Methodology

### 3.1 Research design

This study adopted a research and development (R&D) design operationalized through the ADDIE model, combined with a quasi-experimental pre-test/post-test control group design to evaluate effectiveness. The R&D approach was selected because the primary goal was to produce, validate, and empirically evaluate an instructional product — a purpose for which purely experimental or descriptive designs are insufficient ( Borg & Gall, 1983; Branch, 2009). The quasi-experimental component was necessitated by the logistical constraints of natural classroom groupings, which precluded random assignment of individual students to conditions.

### 3.2 Participants

The study population comprised Grade XI students enrolled in senior high schools (SMA/SMK) located in coastal subdistricts across three districts in West Java Province, Indonesia. Purposive sampling was employed to select four schools that met three criteria: (1) geographic proximity to a coastal ecosystem (< 5 km from shoreline), (2) availability of basic digital infrastructure (minimum one student per device), and (3) alignment of school curricula with the national geography competency framework. A total of 148 students (74 experimental, 74 control) participated across the four schools. Prior to implementation, equivalence between experimental and control groups was verified through an independent-samples t-test on pre-test scores (t(146) = 0.43, p = .67), confirming no significant baseline differences. Expert validators included four subject-matter experts in coastal geography and environmental science and three instructional technology specialists.

### 3.3 Development of ESD-CoastalDTM

Development followed the five ADDIE phases. In the Analysis phase, a needs assessment was conducted through curriculum document analysis, student diagnostic tests (n = 30, non-participants), and teacher interviews (n = 8) to identify learning gaps and content priorities. The Design phase produced a learning blueprint specifying six thematic modules: (1) coastal ecosystem structure and biodiversity; (2) biogeochemical cycles in marine environments; (3) anthropogenic pressures and ecological degradation; (4) climate change and coastal vulnerability; (5) community-based coastal management; and (6) geospatial tools for coastal monitoring. Each module was structured around ESD competency anchors: systems thinking, anticipatory thinking, normative thinking, strategic thinking, and collaborative action ( UNESCO, 2017).

In the Development phase, materials were produced as interactive digital e-modules incorporating embedded videos, interactive maps, annotated satellite imagery, gamified comprehension checks, and reflection prompts aligned with ESD dispositions. The platform used was a responsive web-based application compatible with mobile devices, given the connectivity constraints of coastal school environments. The Implementation phase involved a six-week classroom deployment in the experimental schools, with the control group receiving standard textbook-based instruction on the same content. The Evaluation phase encompassed both formative evaluation (expert validation, small-group pilot with n = 15) and summative evaluation (full quasi-experiment).

### 3.4 Instruments

Geographic literacy was assessed using the Geographic Literacy Scale for Coastal Secondary Students (GLS-CSS), developed and validated for this study. The GLS-CSS comprises 40 items distributed across four subscales: Spatial Thinking (10 items), Environmental Knowledge (10 items), Geospatial Skills (10 items), and Sustainability Orientation (10 items). Item development followed a three-stage process: expert item generation, cognitive interviewing (n = 12), and confirmatory factor analysis (CFA) using a calibration sample (n = 95, non-participants). The final instrument demonstrated acceptable model fit (CFI = 0.94, RMSEA = 0.058, SRMR = 0.062) and strong internal consistency across subscales (α = .83 to.91). Content validity was established through expert review using the Content Validity Index (CVI ≥ .80 for all items). DTM validity was assessed using a 5-domain expert rating scale covering instructional content accuracy, ESD integration fidelity, media design quality, navigational functionality, and contextual relevance.

### 3.5 Data analysis

DTM validity was quantified using the mean validity coefficient per domain and overall, with acceptable validity defined as M ≥ 3.40/4.00. Geographic literacy outcome analyses employed analysis of covariance (ANCOVA) with pre-test scores as covariates, controlling for initial group differences. Cohen’s d was computed to quantify effect sizes. Subscale-level analyses were conducted using separate univariate ANCOVAs with Bonferroni correction for multiple comparisons (α = .0125 per test). All analyses were performed in IBM SPSS Statistics 27.0. Ethical approval was obtained from the institutional review board, and written informed consent was secured from all student participants and their guardians.

## 4. Results

### 4.1 Expert validity of ESD-CoastalDTM

Table 1 summarizes the expert validity ratings across the five DTM evaluation domains. The overall validity coefficient was M = 3.79 (SD = 0.12), exceeding the threshold for high validity (≥ 3.40). Content accuracy received the highest rating (M = 3.82), reflecting experts’ assessment that the coastal ecology content was scientifically accurate, current, and appropriately scoped for senior secondary level. ESD integration fidelity (M = 3.78) was rated positively, with validators noting that ESD competency anchors were explicitly and consistently embedded across all six modules. Media design quality (M = 3.76) and navigational functionality (M = 3.74) were also rated highly, with minor recommendations addressed during iterative revision cycles. Contextual relevance received a slightly lower but still strong rating (M = 3.69), with validators suggesting greater incorporation of local coastal species and community case studies specific to Indonesian coastal contexts — revisions that were incorporated in the final version.

**Table 1.** Expert validity ratings of ESD-CoastalDTM by domain.

Domain	n Validators	Mean (M)	SD	Validity Category
Content Accuracy	4	3.82	0.09	High
ESD Integration Fidelity	7	3.78	0.11	High
Media Design Quality	3	3.76	0.14	High
Navigational Functionality	3	3.74	0.13	High
Contextual Relevance	7	3.69	0.18	High
Overall	7	3.79	0.12	High

### 4.2 Effects on geographic literacy

Prior to intervention, no significant difference in geographic literacy scores was observed between the experimental (M = 48.3, SD = 7.1) and control groups (M = 48.8, SD = 6.9; t(146) = 0.43, p = .67). Following the six-week intervention, ANCOVA with pre-test scores as covariate revealed a statistically significant main effect of condition on post-test geographic literacy scores, F(1, 145) = 87.4, p < .001, partial η ^2^ = .376. The experimental group demonstrated substantially higher post-test scores (adjusted M = 72.4, SE = 0.91) relative to the control group (adjusted M = 52.1, SE = 0.91), with a large effect size (Cohen’s d = 0.84, 95% CI [0.61, 1.06]). Table 2 presents the full descriptive and inferential statistics.

**Table 2.** Geographic literacy pre-test and post-test scores by group.

Group	Pre-test M (SD)	Post-test M (SD)	Adjusted M	Cohen’s d	p-value
Experimental (n = 74)	48.3 (7.1)	72.4 (8.3)	72.4	0.84	< .001
Control (n = 74)	48.8 (6.9)	52.1 (7.4)	52.1	0.14	.21

### 4.3 Subscale-level effects

Subscale-level ANCOVAs (Bonferroni-corrected α = .0125) revealed significant intervention effects on all four geographic literacy dimensions ( Table 3). Sustainability Orientation demonstrated the largest effect (d = 0.91, p < .001), consistent with the ESD-integrated design’s explicit emphasis on normative and strategic thinking competencies. Spatial Thinking (d = 0.78) and Environmental Knowledge (d = 0.81) showed large effects attributable to the interactive map activities and annotated satellite imagery embedded in the DTMs. Geospatial Skills demonstrated a moderate-to-large effect (d = 0.71), reflecting students’ engagement with the coastal monitoring simulation tasks — though this subscale also showed the greatest variance in the experimental group (SD = 3.4), suggesting differential skill development that warrants further investigation.

**Table 3.** Subscale-level ancova results for geographic literacy dimensions.

Subscale	Exp. Post M (SD)	Ctrl Post M (SD)	F(1,145)	p	d
Spatial Thinking	18.2 (2.7)	12.8 (2.9)	61.4	< .001	0.78
Environmental Knowledge	18.8 (2.6)	13.1 (2.8)	67.9	< .001	0.81
Geospatial Skills	17.1 (3.4)	12.3 (2.6)	55.2	< .001	0.71
Sustainability Orientation	18.3 (2.5)	13.9 (2.4)	78.1	< .001	0.91

## 5. Discussion

### 5.1 Validity and design quality of ESD-CoastalDTM

The high expert validity ratings obtained across all five evaluation domains (overall M = 3.79/4.00) affirm that the ADDIE-guided development process produced instructional materials that are scientifically credible, pedagogically coherent, and technically functional. The strong content validity rating (M = 3.82) reflects the integration of current coastal ecology literature into module content, ensuring alignment with scientific consensus on mangrove ecology, coral bleaching dynamics, and coastal erosion processes. The ESD integration fidelity rating (M = 3.78) is particularly noteworthy, as it indicates that subject-matter experts recognized the ESD competency anchors as meaningfully embedded rather than superficially appended — a distinction that prior work on ESD-labeled curricula has shown to be critical for authentic transformative learning ( Wals, 2012; Rieckmann, 2018). The comparatively lower contextual relevance score (M = 3.69) — while still high — signals that material developers in archipelagic contexts should prioritize hyperlocal ecological case studies and species data over generic tropical coastal exemplars, a recommendation that aligns with place-based education theory ( Kudryavtsev et al., 2012).

### 5.2 Geographic literacy development

The large and statistically significant effect of ESD-CoastalDTM on overall geographic literacy ( *d* = 0.84) substantially exceeds the effect sizes typically reported in general geography technology-enhanced learning interventions ( *d* = 0.30–0.55; Patterson et al., 2019; Kerski, 2013). Three potential mechanisms may account for this elevated effect. First, the place-based ecological embedding of all six modules — situating content within students’ own coastal environments — activated prior contextual knowledge and generated a motivational salience that generic geography DTMs lack. This interpretation is consistent with Kudryavtsev et al.’s (2012) place attachment theory, which predicts heightened environmental learning when instructional stimuli directly reference the learner’s inhabited landscape.

Second, the ESD-competency scaffolding — particularly the explicit integration of systems thinking and anticipatory thinking tasks — may have engaged higher-order cognitive processing that deepened encoding and transfer of geographic concepts. This aligns with Bloom’s revised taxonomy as applied to geographic education ( Jo & Bednarz, 2009) and with Brundiers et al.’s (2021) evidence that ESD-structured curricula outperform content-only approaches on transfer tasks. Third, the interactive digital affordances of the platform — including dynamic map manipulation, satellite imagery annotation, and gamified self-assessment — provided multimodal engagement pathways that are theoretically linked to deeper geographic reasoning development ( Bednarz et al., 2013; Michail et al., 2007). Notably, the absence of an active-control condition — one employing an alternative digital curriculum without ESD framing — precludes attribution of the observed effects exclusively to ESD scaffolding versus digital modality per se; future studies should include such a control to isolate the pedagogical contribution of the ESD design ( Bednarz et al., 2013; Michail et al., 2007).

### 5.3 Differential effects across geographic literacy dimensions

The finding that Sustainability Orientation showed the largest effect (d = 0.91) — exceeding Spatial Thinking (d = 0.78), Environmental Knowledge (d = 0.81), and Geospatial Skills (d = 0.71) — merits theoretical attention. This pattern suggests that sustainability disposition is more responsive to short-duration, contextually embedded ESD interventions than previously assumed. The conventional view in the ESD literature has been that normative orientation changes slowly, requiring longitudinal exposure to sustainability values frameworks ( Boeve-de Pauw et al., 2015; Osman et al., 2021). The present findings tentatively challenge this assumption, suggesting that short-duration, place-based ESD interventions may produce large changes in sustainability orientation when the instructional context directly engages students’ lived ecological realities; however, causal claims are constrained by the absence of randomisation and long-term follow-up. The relatively lower effect on Geospatial Skills The relatively lower effect on Geospatial Skills (d = 0.71) and its higher within-group variance (SD = 3.4) suggests that technical geospatial competencies require more differentiated scaffolding and extended practice time — an implication relevant to future DTM design iterations.

### 5.4 Limitations

Several methodological limitations constrain the generalizability and causal interpretability of these findings. First, the quasi-experimental design, while statistically controlling for pre-test differences via ANCOVA, cannot fully eliminate selection bias; unobserved confounds such as teacher motivation, classroom climate, and prior technology exposure may have differentially influenced outcomes. The design does not support causal inference in the strict sense — findings should be interpreted as preliminary evidence pending replication with randomised assignment. Second, novelty effects (Hawthorne effect) cannot be excluded: students in the experimental group were exposed to a visually immersive digital platform for the first time, which may have inflated engagement and motivation irrespective of ESD content. Third, the absence of an active control condition — a comparator group receiving an alternative DTM without ESD framing — prevents attribution of effects specifically to the ESD scaffolding versus digital modality per se. Fourth, the GLS-CSS instrument was validated on a pilot sample of n = 95, which yields marginal power for CFA (CFI = 0.94 is borderline acceptable); independent large-sample replication of the measurement model is recommended before the instrument is adopted in other contexts. Fifth, the six-week implementation window is likely insufficient to assess behavioural sustainability outcomes, which ESD theory positions as the ultimate goal of transformative environmental education. Sixth, the sample was drawn exclusively from coastal communities in West Java, limiting direct generalizability to other Indonesian coastal contexts (e.g., Kalimantan, Sulawesi, eastern archipelago regions) where ecological conditions, socioeconomic profiles, and digital infrastructure differ substantially. Future research employing randomised controlled trials with active control conditions and longer follow-up periods across multiple coastal provinces would substantially strengthen the evidence base.

## 6. Conclusion

This study developed, validated, and evaluated ESD-CoastalDTM — a set of six-module digital teaching materials on coastal ecology integrating Education for Sustainable Development competencies — and demonstrated their effectiveness in enhancing geographic literacy among senior high school students in coastal communities. Expert validation confirmed high material quality across content accuracy, ESD integration, media design, and contextual relevance. A quasi-experimental evaluation revealed a large positive effect on overall geographic literacy (d = 0.84), with the sustainability orientation dimension showing the strongest responsiveness to the intervention (d = 0.91).

These findings contribute to geographic literacy theory by establishing a place-based, ESD-integrated mechanism for simultaneous development across all four geographic literacy dimensions. They contribute methodologically by introducing and validating the GLS-CSS instrument for use in coastal secondary education research. Practically, the ADDIE-structured development process provides a replicable blueprint for educational technology developers and curriculum policymakers in archipelagic nations seeking to bridge coastal ecology content with sustainability education mandates. Future research should extend this work through longitudinal designs, multi-provincial samples, and behavioral outcome measures to fully assess the transformative potential of ESD-integrated coastal geography education.

## Ethics statement

This study did not require formal Institutional Review Board approval under Indonesian educational research guidelines because: (i) the study constituted standard educational practice (curriculum material evaluation) and did not involve experimental medical, psychological, or invasive procedures; (ii) all student responses were anonymous and unlinked to any personally identifiable information; and (iii) participation was voluntary with written informed consent obtained from school principals and district education authorities. Research permissions were obtained from the principals of the participating schools and the relevant District Education Office (Dinas Pendidikan) prior to data collection (Reference: No. 421/235/SMAN.4-TTE/2026). All procedures were conducted in accordance with the Declaration of Helsinki principles for educational research involving minors.

## AI disclosure

AI-assisted tools were used for language refinement only. All intellectual content, research design, analysis, interpretation, and conclusions were developed independently by the authors.

## Data Availability

The data supporting the findings of this study are publicly available in the Zenodo open repository. The dataset includes anonymised GLS-CSS pre- and post-test scores, ANCOVA results, validity ratings, and instrument specifications. It does not contain any personally identifiable information. Citation: Muhammad, I. (2026). Dataset: ESD-Integrated Digital Teaching Materials for Coastal Ecology — Geographic Literacy Study (Muhammad, I et al., 2026) [Data set]. Zenodo. https://doi.org/10.5281/zenodo.20569465.
