# ESD-Integrated Digital Learning for Geographic Literacy in Coastal Secondary Schools: Development and Evaluation

**DOI:** 10.12688/f1000research.184677.2

**Published:** 2026-10-26

**Authors:** Muhammad Ikhsan, Epon Ningrum, Mamat Ruhimat, Iwan Setiawan, Sony Nugratama Hijrawad, Irwan Abdullah, Salman Alparis Sormin, Tuti Purwati, Dwi Nanda Akhmad Romadhon

**Affiliations:** 1 Universitas Pendidikan Indonesia, Bandung, West Java, Indonesia

**Keywords:** coastal ecology education; digital teaching materials; Education for Sustainable Development; geographic literacy; ADDIE model; secondary education

## Abstract

**Background:**

Coastal ecosystems are among the most productive and socioeconomically important environments globally. However, students in coastal communities often exhibit limited geographic literacy regarding the ecological processes that sustain these ecosystems. Contextualized digital learning resources integrating sustainability principles may help address this challenge.

**Objective:**

This study aimed to develop, validate, and evaluate the effectiveness of ESD-integrated digital teaching materials (DTMs) on coastal ecology in improving geographic literacy among senior high school students in coastal areas.

**Method:**

This research and development (R&D) study employed the ADDIE model and a quasi-experimental pre-test–post-test control group design. Participants were 148 Grade XI students from four coastal-area schools selected through purposive sampling. The DTMs were evaluated by four subject-matter experts and three instructional technology specialists. Geographic literacy was assessed using a 40-item instrument covering spatial thinking, environmental knowledge, geospatial skills, and sustainability orientation. The experimental group used ESD-integrated DTMs, whereas the control group received conventional textbook-based instruction.

**Results:**

The DTMs demonstrated high validity, with mean scores of 3.82/4.00 for content and 3.76/4.00 for media quality. Students in the experimental group showed a significant improvement in geographic literacy following the intervention (d = 0.84, p < .001), while the control group exhibited no significant change (p = .21). Among the assessed dimensions, sustainability orientation recorded the strongest effect (d = 0.91).

**Conclusion:**

The findings indicate that ESD-integrated DTMs are a promising approach to supporting geographic literacy in coastal secondary education. However, because the quasi-experimental comparison involved digital materials in the experimental group and conventional textbooks in the control group, the independent contribution of ESD integration cannot be separated from the effects of digital modality and instructional novelty. Longitudinal studies are needed to determine whether the observed improvements, particularly in sustainability orientation, persist over time and translate into sustained environmental knowledge, attitudes, and behaviors.

## 1. Introduction

Coastal ecosystems, including mangroves, coral reefs, seagrass beds, and estuaries, provide essential ecological functions and support human well-being through food provision, climate regulation, biodiversity conservation, and livelihood generation ( Douglas, 2017). These ecosystems are particularly important in archipelagic countries such as Indonesia, where coastal environments are closely connected to community livelihoods, regional development, and environmental sustainability. However, geographical proximity to coastal ecosystems does not necessarily ensure that students understand the ecological processes, spatial relationships, and human activities that influence these environments. This challenge highlights the need for geography education that connects scientific concepts with local environmental conditions and enables students to interpret the relationships between spatial patterns, ecosystem processes, and human activities. In Indonesia, this need is also relevant to the ongoing implementation of the Merdeka Curriculum in geography education, which presents opportunities for contextualized and innovative learning while requiring attention to practical implementation challenges ( Sobirin, 2024).

Geographic literacy is an important educational outcome because it involves more than recalling geographical facts; it requires learners to interpret spatial information, understand geographical relationships, and apply geographical knowledge to real-world problems. Recent research indicates that geographical thinking encompasses several related but conceptually distinct forms of reasoning, including spatial thinking, geographical reasoning, and systems thinking ( Bendl et al., 2025). For the purposes of this study, geographic literacy is operationalized through four complementary dimensions: spatial thinking, environmental knowledge, geospatial skills, and sustainability orientation. Spatial thinking concerns the interpretation of locations, distributions, patterns, and spatial relationships. Environmental knowledge involves understanding ecological processes and interactions between human and natural systems. Geospatial skills concern the interpretation and use of maps, spatial data, and geographical representations. Sustainability orientation refers to learners’ reported awareness and dispositions concerning environmental responsibility and sustainable decision-making. Although these dimensions are interconnected, they are not interchangeable: understanding environmental processes does not automatically confer the ability to interpret spatial data, and increased sustainability awareness does not necessarily translate into sustained environmental behavior. Accordingly, an instructional approach intended to support geographic literacy needs to integrate geographical knowledge, spatial reasoning, geospatial representation, and reflection on sustainability-related decisions.

Education for Sustainable Development (ESD), particularly as articulated in UNESCO’s *Education for Sustainable Development Goals: Learning Objectives*, provides a relevant pedagogical framework for connecting these learning dimensions ( UNESCO, 2017). ESD emphasizes the development of competencies that enable learners to understand complex sustainability challenges and respond to them through informed and responsible action. Among these competencies, systems thinking and anticipatory thinking are particularly relevant to coastal ecology education. Systems thinking enables learners to identify relationships among environmental, social, and economic components, whereas anticipatory thinking supports the consideration of possible futures, risks, and the consequences of alternative decisions. Research on sustainability competencies has emphasized the importance of explicitly incorporating these competencies into educational design ( Brundiers et al., 2021). Applied to coastal ecology, systems thinking can guide students in examining how coastal land use, ecosystem degradation, human livelihoods, and environmental management interact across spatial scales. Anticipatory thinking can further encourage students to consider how present decisions may influence future ecosystem conditions, coastal vulnerability, and community well-being. Evidence from climate-change education also illustrates the relevance of contextualized problem scenarios for examining systems-thinking competencies and their relationship with subject knowledge ( Liu, 2023). Together, these perspectives provide a theoretical mechanism through which ESD-oriented learning may support geographic literacy: students are encouraged not only to identify geographical features but also to explain interdependencies, interpret environmental change, and evaluate the potential consequences of sustainability-related decisions.

Digital teaching materials (DTMs) can provide opportunities to represent complex geographical phenomena through interactive maps, annotated satellite imagery, multimedia explanations, and other spatial visualizations. Nevertheless, the educational value of digital materials depends not merely on their technological features but also on the learning processes they are designed to support. Standard digital geography resources may present maps, videos, images, and factual information without necessarily requiring learners to connect system components, examine temporal changes, compare future scenarios, or justify sustainability-related decisions. ESD-integrated DTMs can address this pedagogical distinction by organizing digital resources around explicit sustainability competencies. For example, systems-oriented activities can ask students to connect coastal ecosystem components with human activities; anticipatory tasks can prompt them to consider the possible consequences of coastal development or ecosystem degradation; and reflective decision-making activities can require them to evaluate alternative management strategies. These learning processes offer a theoretical rationale for integrating ESD into digital materials rather than treating digital interactivity as an educational innovation in itself. Empirical research on systems thinking and simulation in sustainability education provides relevant evidence that these pedagogical elements can be investigated through structured learning interventions ( Green et al., 2021). More broadly, educational technology research emphasizes that digital innovation should be evaluated in relation to learning quality, inclusion, and educational outcomes rather than technological adoption alone ( Costa et al., 2024). However, these studies do not establish that ESD-integrated digital materials are inherently superior to all conventional or digital alternatives; the relative contribution of pedagogical design and digital delivery requires appropriate empirical comparison.

Place-based learning offers an additional rationale for contextualizing digital materials around local coastal ecosystems. By connecting geographical concepts with environments familiar to learners, place-based approaches can make abstract environmental processes more relevant to students’ everyday experiences. Recent research on place-based ocean education has explored how locally contextualized activities can support students’ understanding of, and connections with, marine environments ( Freitas et al., 2026). In the present study, coastal ecology therefore serves not merely as a content topic but as a context for connecting spatial interpretation, ecological explanation, and sustainability-related reflection. This contextualization is particularly relevant to coastal secondary schools because learning materials can relate geographical concepts to the environmental conditions and community issues that students may encounter in their surrounding areas. Nevertheless, contextual relevance should be understood as a design rationale rather than as proof that all students possess strong place attachment or that contextualization alone guarantees improved learning outcomes.

Despite the conceptual relevance of ESD, digital learning, and place-based education, the development and empirical evaluation of digital teaching materials that explicitly integrate coastal ecological content, ESD competencies, and multidimensional geographic literacy within a single secondary-school intervention remains an important area for further investigation. Research on geographical thinking has identified diverse conceptualizations of geographical reasoning, while sustainability-education research has emphasized the importance of developing competencies for understanding complex environmental challenges ( Bendl et al., 2025; Brundiers et al., 2021). Research on digital sustainability learning has also examined systems thinking and simulation as components of structured educational interventions ( Green et al., 2021). However, the extent to which these perspectives can be translated into a contextualized digital instructional package for coastal secondary students, evaluated alongside conventional textbook-based instruction, and assessed through a multidimensional geographic literacy instrument warrants further empirical examination. This need is relevant to Indonesian geography education, including the implementation of the Merdeka Curriculum, where locally relevant learning materials may provide opportunities to connect geographical competencies with environmental and sustainability challenges ( Sobirin, 2024). The present study addresses this research need without assuming that digital delivery is inherently more effective than conventional instruction or that the effects of ESD can be isolated from those of the digital medium.

To address this need, the study developed, validated, and evaluated ESD-integrated digital teaching materials on coastal ecology, hereafter referred to as ESD-CoastalDTM, using the ADDIE instructional design model. Three research questions guided the study: (1) To what extent do the developed DTMs meet expert validity criteria for content accuracy and media quality? (2) How do geographic literacy outcomes change following participation in the ESD-CoastalDTM intervention compared with conventional textbook-based instruction? (3) Which dimensions of geographic literacy demonstrate the greatest responsiveness during the intervention?

This study offers three specific contributions. First, it advances content contextualization by organizing digital learning around coastal ecosystems and the relationships between ecological processes and human activities in coastal communities. Second, it integrates ESD competencies, particularly systems thinking and anticipatory thinking, into digital learning activities intended to connect spatial interpretation, environmental understanding, geospatial reasoning, and sustainability-related reflection. Third, it develops and provides an initial psychometric evaluation of the Geographic Literacy Scale for Coastal Secondary Students (GLS-CSS), a 40-item instrument covering spatial thinking, environmental knowledge, geospatial skills, and sustainability orientation. Together, these contributions provide a context-specific example of ESD-integrated digital-material development and an empirical basis for further investigation in coastal secondary education. Because the study employed a quasi-experimental design in which the experimental group received ESD-integrated digital materials and the control group received conventional textbook-based instruction, the findings are interpreted as evidence concerning the combined instructional intervention. The design does not isolate the independent contribution of ESD integration from the effects of digital modality or instructional novelty.

## 2. Theoretical framework and literature review

### 2.1 Education for sustainable development as a pedagogical framework

Education for Sustainable Development (ESD) is a pedagogical approach that develops the knowledge, competencies, values, and capacities required to address sustainability challenges ( UNESCO, 2017). Its educational orientation extends beyond environmental knowledge by integrating critical reflection, systems thinking, anticipatory thinking, and consideration of the consequences of human decisions ( Brundiers et al., 2021). In coastal ecology education, systems thinking enables students to examine relationships among ecosystem components, human activities, and environmental processes, while anticipatory thinking encourages them to consider possible future conditions and the consequences of alternative management decisions. These competencies provide a theoretical mechanism for connecting environmental knowledge with geographic literacy by requiring learners to interpret spatial relationships, explain human-environment interactions, and evaluate sustainability-related problems rather than merely recall geographical information.

A critical distinction in ESD research is that changes in knowledge, attitudes, intentions, and self-reported dispositions do not necessarily demonstrate sustained behavioral transformation. Environmental education can influence several of these outcomes, but their magnitude and persistence depend on intervention characteristics, context, and measurement methods ( Ardoin et al., 2020; van de Wetering et al., 2022). Accordingly, sustainability orientation in this study is conceptualized as initial dispositional awareness, reflecting students reported environmental awareness and orientation toward sustainable decision-making at the time of measurement. It is not interpreted as evidence of permanent value change or sustained pro-environmental behavior. A six-week intervention can indicate short-term changes in reported orientation, whereas claims about behavioral persistence require longitudinal assessment and, where feasible, behavioral indicators beyond self-report measures. Furthermore, because the present study compares ESD-integrated digital materials with conventional textbook-based instruction, it evaluates the combined instructional intervention rather than isolating the independent contribution of ESD from digital modality or instructional novelty.

### 2.2 Geographic literacy: Dimensions and measurement

This study Geographic literacy encompasses the ability to interpret spatial information, understand environmental processes, apply geospatial representations, and relate geographical knowledge to real-world challenges ( Solem, 2020; Susan Huynh, 2013). In this study, it is operationalized through four dimensions: spatial thinking, environmental knowledge, geospatial skills, and sustainability orientation. Spatial thinking supports the interpretation of location, distribution, and spatial relationships; environmental knowledge enables explanations of ecological processes and human-environment interactions; geospatial skills concern the interpretation and use of maps and other spatial representations; and sustainability orientation reflects students’ reported dispositions toward environmental responsibility. These dimensions are related but not interchangeable. In particular, stronger environmental awareness does not necessarily indicate improved technical geospatial performance or sustained environmental behavior.

Place-Based Education (PBE) provides a pedagogical rationale for connecting geographical learning with the ecological, social, and cultural characteristics of students’ local environments. Through contextualized inquiry and engagement with meaningful local issues, PBE can help students relate geographical concepts to environmental processes and community experiences ( Gruenewald, 2003; Yemini et al., 2025). Place attachment, however, is a distinct construct referring to emotional and functional bonds with particular places. Kudryavtsev et al. (2012) distinguish place attachment from place meaning and demonstrate that environmental education may influence these dimensions differently. In the present study, PBE and place attachment provide contextual and theoretical rationales for using coastal ecosystems as learning contexts, rather than additional measured outcomes. Because the GLS-CSS does not directly assess place attachment, the study cannot establish that the intervention strengthened students’ attachment to coastal places. Similarly, sustainability orientation is interpreted as initial dispositional awareness, not permanent behavioral transformation; determining whether such changes persist requires longitudinal follow-up and, where possible, direct behavioral assessment.

### 2.3 Digital teaching materials in geography education

This study Digital teaching materials (DTMs) are structured technology-mediated resources designed to support specified learning objectives, including electronic modules, interactive multimedia, digital maps, and spatial visualizations. In geography education, these resources can facilitate the interpretation of spatial patterns, visualization of environmental processes, and exploration of geographical information. Nevertheless, the presence of digital features does not automatically produce higher-order geographical reasoning. The educational value of DTMs depends on how their content, representations, and learning tasks are aligned with the intended competencies. Digital resources that primarily present information may support content access without necessarily requiring students to connect system components, examine environmental change, or evaluate sustainability-related decisions.

The ESD-CoastalDTM developed in this study integrates coastal ecological content with learning activities informed by systems thinking, anticipatory thinking, and sustainability-oriented reflection. Interactive representations can support the examination of relationships among coastal ecosystems, human activities, and environmental pressures, while structured questions can encourage students to consider future consequences and alternative management decisions. The integration of PBE further situates these activities within a geographically meaningful coastal context. This design provides a theoretical rationale for the intervention, but its effectiveness must be established empirically. Because the control group received conventional textbook-based instruction rather than an active digital comparison, the study cannot isolate the effects of ESD pedagogy from digital modality, novelty, or differences in learning engagement.

### 2.4 The ADDIE model as a development framework

This study The ADDIE model-Analysis, Design, Development, Implementation, and Evaluation-provides a systematic framework for developing and evaluating instructional products ( Branch, 2010; Peterson, 2003). Its phases support alignment between identified learning needs, instructional objectives, material design, expert validation, classroom implementation, and outcome evaluation. In this study, the Analysis phase identified learning needs and relevant coastal ecology content; the Design phase specified learning objectives and ESD competencies; the Development phase translated these specifications into digital learning materials; and the Implementation and Evaluation phases supported classroom use, expert review, and assessment of learning outcomes.

The theoretical frameworks and methodological model serve complementary functions: ESD defines the sustainability competencies informing the instructional activities, PBE provides the rationale for contextualizing content within local coastal environments, and ADDIE structures the development and evaluation process. The GLS-CSS assesses four dimensions of geographic literacy, while its psychometric evidence must be interpreted in accordance with the available validation data. In particular, the CFA calibration sample of 95 participants is relatively small for a 40-item instrument; therefore, model-fit and internal-consistency results should be regarded as preliminary evidence rather than definitive confirmation of the factor structure. Likewise, the quasi-experimental evaluation supports conclusions about outcomes associated with the combined ESD-integrated digital intervention compared with textbook-based instruction, but does not establish the independent causal contribution of ESD or demonstrate long-term behavioral change.

## 3. Methodology

### 3.1 Research design

This study adopted a research and development (R&D) design operationalized through the ADDIE model, combined with a quasi-experimental pre-test/post-test control group design to evaluate effectiveness. The R&D approach was selected because the primary goal was to produce, validate, and empirically evaluate an instructional product ( Branch, 2009, 2010; Peterson, 2003). The quasi-experimental component was necessitated by the logistical constraints of natural classroom groupings, which precluded random assignment of individual students to conditions. The intervention was evaluated as an integrated package combining ESD-oriented coastal ecology content, interactive digital features, and associated learning activities, whereas the control group received conventional printed textbook instruction covering the same general content. An active digital control group was not included because this development-stage study prioritized evaluating the integrated product against conventional instruction. Consequently, the findings cannot isolate the contribution of ESD pedagogy from digital modality, instructional novelty, or differential learner engagement. Pre-test/post-test assessment does not independently eliminate potential Hawthorne effects; future studies should include an equally interactive digital control condition to distinguish pedagogical effects from those of digital delivery ( Adair et al., 1989; Tsay et al., 2020).

### 3.2 Participants

The study population comprised Grade XI students enrolled in senior high schools (SMA/SMK) located in coastal subdistricts across three districts in West Java Province, Indonesia. Purposive sampling was employed to select four schools that met three criteria: (1) geographic proximity to a coastal ecosystem (< 5 km from shoreline), (2) availability of basic digital infrastructure (minimum one student per device), and (3) alignment of school curricula with the national geography competency framework. A total of 148 students (74 experimental, 74 control) participated across the four schools. Baseline pre-test scores did not differ significantly between groups, t (146) = 0.43, p = 0.67. Expert validators comprised four coastal geography and environmental science experts and three instructional technology specialists. The non-significant baseline test should not be interpreted as proof of group equivalence or as evidence that all confounding variables were controlled. Because students were recruited from intact classes across four schools, school and teacher characteristics may have influenced the outcomes. The manuscript should therefore specify how schools and classes were allocated to each condition and report the number of schools per group. If conditions were implemented in different schools, school-level differences may be confounded with the intervention and cannot be reliably separated with only four schools.

Moreover, School-level permission and participant consent procedures were implemented in accordance with the documentation available for the study. Written informed consent was obtained from parents or legal guardians, and student assent was obtained where applicable. Participant confidentiality was maintained throughout data collection and analysis. These permissions and consent procedures should not be interpreted as formal ethics committee approval. The need for institutional ethics review will be clarified with the relevant institution and journal.

### 3.3 Development of ESD-CoastalDTM

Development followed the five ADDIE phases. In the Analysis phase, a needs assessment was conducted through curriculum document analysis, student diagnostic tests (n = 30, non-participants), and teacher interviews (n = 8) to identify learning gaps and content priorities. The Design phase produced a learning blueprint specifying six thematic modules: (1) coastal ecosystem structure and biodiversity; (2) biogeochemical cycles in marine environments; (3) anthropogenic pressures and ecological degradation; (4) climate change and coastal vulnerability; (5) community-based coastal management; and (6) geospatial tools for coastal monitoring. Each module was structured around ESD competency anchors: systems thinking, anticipatory thinking, normative thinking, strategic thinking, and collaborative action. During Development, interactive web-based e-modules incorporated videos, interactive ( UNESCO, 2017) e maps, annotated satellite imagery, comprehension checks, and reflection prompts. The Implementation phase involved six weeks of classroom instruction, with the control group receiving textbook-based instruction on the same general content. Formative evaluation included expert validation and a small-group pilot involving 15 students; summative evaluation used the quasi-experimental study. To support implementation fidelity, the study should document teacher preparation, planned instructional time, module sequence, and procedures for monitoring classroom delivery. If no standardized teacher orientation or formal fidelity monitoring was conducted, this should be acknowledged as a limitation because teacher enthusiasm, familiarity with digital tools, and implementation differences may have affected outcomes ( Carroll et al., 2007).

### 3.4 Instruments

Geographic literacy was assessed using the Geographic Literacy Scale for Coastal Secondary Students (GLS-CSS), developed and validated for this study. The 40-item instrument comprised four subscales: Spatial Thinking, Environmental Knowledge, Geospatial Skills, and Sustainability Orientation, each containing 10 items. Item development involved expert review, cognitive interviewing with 12 students, and confirmatory factor analysis (CFA) using a separate calibration sample of 95 non-participating students. The model showed acceptable fit (CFI = 0.94, RMSEA = 0.058, SRMR = 0.062), with internal consistency coefficients ranging from α = 0.83 to 0.91. Content validity was assessed using the Content Validity Index (CVI ≥ .80 for all items). DTM validity was evaluated by experts across five domains: content accuracy, ESD integration, media design, navigation, and contextual relevance. The CFA sample of n = 95 provides preliminary calibration evidence rather than definitive psychometric validation for a 40-item, four-factor instrument. CFA sample-size adequacy depends on model complexity, factor loadings, and indicator characteristics rather than a universal respondent-to-item ratio ( Wolf et al., 2013). Accordingly, the reported fit indices and reliability estimates should be interpreted cautiously and replicated in a substantially larger, independent sample to examine the stability and generalizability of the proposed structure.

### 3.5 Data analysis

The study DTM validity was summarized using mean expert ratings for each domain and overall, with acceptable validity defined as M ≥ 3.40 on a four-point scale. Geographic literacy outcomes were analyzed using analysis of covariance (ANCOVA), with post-test scores as dependent variables, pre-test scores as covariates, and instructional condition as the independent variable. Cohen’s d was calculated to quantify effect sizes. Separate univariate ANCOVAs were conducted for the four subscales with Bonferroni correction (α = .0125). Analyses were performed using IBM SPSS Statistics 27.0. ANCOVA assumptions, including homogeneity of regression slopes, should be assessed and reported. Adjusted group means, effect estimates, confidence intervals, and the definition of Cohen’s d should be provided where available. Because participants were nested within intact classes and four schools, potential clustering should be considered when interpreting standard errors and statistical significance. A significant pre–post change in one group combined with a non-significant change in the other does not itself establish a significant between-group effect; conclusions should be based on the direct comparison between conditions.

### 3.6 Methodological limitations

Several methodological limitations should be considered. First, the study compared ESD-integrated digital teaching materials with conventional textbook instruction without an active digital control group. Thus, observed outcomes reflect the combined intervention package and cannot be attributed exclusively to ESD pedagogy; digital modality, instructional novelty, learner engagement, and teacher-related factors remain alternative explanations. Future studies should compare the intervention with an equally interactive digital condition while controlling instructional content, time, and teacher attention. Second, the CFA used a calibration sample of 95 students for a 40-item instrument. Although the fit and reliability estimates were encouraging, they represent preliminary evidence requiring replication with a larger independent sample ( Wolf et al., 2013).

Third, the use of intact classes across four schools may leave school-level and teacher-level influences insufficiently controlled, particularly if each condition was implemented in different schools. Future research should document teacher preparation and implementation fidelity and use designs that better distinguish school, teacher, modality, and pedagogical effects. Finally, the six-week intervention does not establish the long-term persistence of geographic literacy gains or sustainability-related dispositions. Longitudinal follow-up and behavioral measures are needed to determine whether short-term outcomes translate into sustained understanding and action.

## 4. Results

### 4.1 Expert validity of ESD-CoastalDTM

Expert validation was conducted to evaluate the scientific accuracy, pedagogical integration of Education for Sustainable Development (ESD), media quality, usability, and contextual relevance of ESD-CoastalDTM. The evaluation involved subject-matter experts and instructional technology specialists, with ratings assessed against a predefined acceptance threshold of 3.40 on a four-point scale. This multidimensional evaluation was intended not only to establish the initial suitability of the materials but also to identify aspects requiring refinement before classroom implementation. The results are presented in Table 1.

**Table 1.** Expert validity ratings of ESD-CoastalDTM by domain.

Domain	n Validators	Mean (M)	SD	Validity Category
Content Accuracy	4	3.82	0.09	High
ESD Integration Fidelity	7	3.78	0.11	High
Media Design Quality	3	3.76	0.14	High
Navigational Functionality	3	3.74	0.13	High
Contextual Relevance	7	3.69	0.18	High
Overall	7	3.79	0.12	High

As shown in Table 1, the validation results indicate that ESD-CoastalDTM was considered suitable for further classroom evaluation, while also identifying contextualization as an area for refinement. The comparatively greater variability in ratings for Contextual Relevance (SD = 0.18) suggests that expert assessments of how effectively local coastal characteristics and community contexts were represented were less consistent than their assessments of other domains. This finding highlights the importance of ensuring that digital learning materials connect scientific concepts with locally meaningful environmental conditions and community experiences. Accordingly, the validation process informed revisions to strengthen the representation of local coastal species and community-based case studies. These findings support the use of expert validation as a basis for refining the instructional product, although they do not by themselves demonstrate improvements in students’ geographic literacy.

### 4.2 Effects on geographic literacy

Geographic literacy was assessed before and after the six-week intervention to examine changes in the experimental and control groups. Baseline comparisons were conducted to identify potential initial differences, while analysis of covariance (ANCOVA) was used to compare post-test outcomes after adjusting for pre-test scores. The analysis indicated a statistically significant effect of instructional condition on post-test geographic literacy, with higher adjusted scores in the experimental group than in the control group. Table 2 presents the descriptive statistics and effect-size estimates for both groups.

**Table 2.** Geographic literacy pre-test and post-test scores by group.

Group	Pre-test M (SD)	Post-test M (SD)	Adjusted M	Cohen’s d	p-value
Experimental (n = 74)	48.3 (7.1)	72.4 (8.3)	72.4	0.84	< .001
Control (n = 74)	48.8 (6.9)	52.1 (7.4)	52.1	0.14	.21

The findings suggest that students who received ESD-integrated digital teaching materials demonstrated greater improvements in geographic literacy than those receiving conventional textbook-based instruction. The pattern provides preliminary evidence that combining coastal ecology content, interactive digital representations, and sustainability-oriented learning activities may support students’ understanding of geographical concepts and their application to environmental issues. Nevertheless, the results should be interpreted as evidence of the effectiveness of the integrated instructional package relative to the textbook-based condition, rather than as proof of the independent effect of ESD pedagogy. Because an active digital control group was not included, the respective contributions of digital modality, instructional novelty, and ESD integration cannot be disentangled.

### 4.3 Subscale-level effects

Subscale-level ANCOVAs were conducted to examine whether the intervention outcomes differed across the four dimensions of geographic literacy, using a Bonferroni-adjusted significance threshold of α = .0125. The analysis revealed statistically significant between-group differences across all four dimensions, although the magnitude of the observed effects varied. Sustainability Orientation showed the largest effect size, followed by Environmental Knowledge, Spatial Thinking, and Geospatial Skills. Table 3 presents the post-test descriptive statistics and inferential results for each dimension.

**Table 3.** Subscale-level ancova results for geographic literacy dimensions.

Subscale	Exp. Post M (SD)	Ctrl Post M (SD)	F(1,145)	p	d
Spatial Thinking	18.2 (2.7)	12.8 (2.9)	61.4	< .001	0.78
Environmental Knowledge	18.8 (2.6)	13.1 (2.8)	67.9	< .001	0.81
Geospatial Skills	17.1 (3.4)	12.3 (2.6)	55.2	< .001	0.71
Sustainability Orientation	18.3 (2.5)	13.9 (2.4)	78.1	< .001	0.91

The variation in effect sizes suggests that the intervention outcomes were not uniform across the four dimensions of geographic literacy. The comparatively large effect for Sustainability Orientation may reflect its explicit alignment with sustainability-related reflection, normative thinking, and strategic decision-making activities in the ESD-integrated modules. In contrast, Geospatial Skills showed the greatest score dispersion in the experimental group (SD = 3.4), compared with SD = 2.5 for Sustainability Orientation. This pattern indicates greater variability in students’ post-test geospatial scores, potentially reflecting differences in prior spatial reasoning, familiarity with digital maps and satellite imagery, or opportunities to practice geospatial tasks. However, these factors were not directly established as explanatory variables in the reported analyses and should therefore be treated as possible explanations rather than confirmed causes. Further research incorporating baseline geospatial proficiency, classroom observations, and student interviews could clarify the sources of this variation and determine whether differentiated scaffolding and additional guided practice would better support students with different starting competencies.

## 5. Discussion

### 5.1 Validity and design quality of ESD-CoastalDTM

The expert validation results provide preliminary evidence that ESD-CoastalDTM achieved a high level of perceived design suitability across the assessed dimensions, with an overall mean rating of 3.79 out of 4.00. The relatively high ratings for Content Accuracy (M = 3.82) and ESD Integration Fidelity (M = 3.78) suggest that the reviewers considered the scientific content and sustainability-oriented learning design appropriate for further classroom evaluation. Nevertheless, these ratings should be interpreted as expert judgments of the product’s quality rather than direct evidence of its effectiveness in improving students’ geographic literacy. This distinction is important because a well-designed instructional product must demonstrate not only scientific accuracy and technical usability but also alignment between learning objectives, instructional activities, and assessment. Accordingly, the validation findings establish a basis for proceeding to classroom evaluation, while the educational value of the materials must be examined separately through evidence of student learning outcomes.

The ESD Integration Fidelity rating also warrants consideration in relation to the broader literature on sustainability education. Integrating ESD into instructional materials involves more than adding sustainability-related topics; it requires meaningful connections between sustainability competencies, disciplinary knowledge, pedagogical activities, and assessment. Nguyen et al. (2022), in their analysis of 429 teacher-education course syllabi, examined the representation of sustainability in learning outcomes, learning content, and didactic approaches, highlighting the importance of considering how these elements are integrated. Similarly, Holst et al. (2024) proposed an approach to monitoring ESD across educational policies, curricula, educator training, and student assessment. In light of this literature, the ESD Integration Fidelity rating of 3.78 suggests that the sustainability-oriented design of ESD-CoastalDTM was positively assessed by experts. However, the rating alone cannot establish whether students experienced the intended depth of sustainability learning or developed the associated competencies. Further evidence from instructional activities, student responses, and assessment tasks would be required to determine how effectively the intended ESD principles were translated into learning experiences.

Contextual Relevance received the lowest mean rating among the five evaluation domains (M = 3.69), although it remained relatively high on the four-point scale. This result is better interpreted as an opportunity for further refinement than as evidence of a substantive design deficiency. ( Chiba et al., 2021), in a systematic review of education for sustainable development and global citizenship education, identified connections between learning and students’ interests, as well as the integration of theoretical and practical learning, as relevant considerations for effective educational experiences. In coastal education, this perspective suggests that contextual relevance involves more than presenting examples of coastal ecosystems; it also requires opportunities for students to connect scientific concepts with environmental conditions and community experiences familiar to them. For ESD-CoastalDTM, the inclusion of local coastal species, ecosystem pressures, and community-based cases may strengthen these connections. However, the expert ratings alone do not establish whether such contextual features improved students’ understanding of local coastal processes. Evidence from students’ analyses of local environmental problems, classroom observations, or interviews would help clarify whether the materials enabled learners to connect geographic knowledge with the conditions of their own coastal environments.

Taken together, the validation results indicate that ESD-CoastalDTM was considered suitable for further classroom evaluation, while also identifying contextual relevance and the depth of ESD integration as dimensions requiring continued attention. The findings contribute to the development of digital teaching materials by demonstrating how scientific content, sustainability-oriented pedagogy, and contextual considerations can be examined together during product development. Nevertheless, the overall validation score should not be treated as a substitute for evidence of instructional effectiveness, nor should the relatively lower contextual relevance score be interpreted as proof that localization was inadequate. Future evaluations could strengthen the evidence base by documenting how expert feedback informs successive revisions, examining the alignment between ESD competencies and assessment tasks, and investigating how students use the materials to interpret authentic coastal issues. Such evidence would provide a more comprehensive assessment of both the design quality of ESD-CoastalDTM and its potential contribution to geographic literacy.

### 5.2 Geographic literacy development

The findings indicate that students who received ESD-CoastalDTM achieved more favorable geographic literacy outcomes than students receiving conventional textbook-based instruction, with a reported overall effect size of d = 0.84. This result suggests a substantial difference between the instructional conditions in the present sample; however, its magnitude should be interpreted in relation to the study design and the characteristics of the comparison condition. Research on geographical thinking emphasizes that geographic literacy encompasses multiple dimensions of knowledge, spatial reasoning, and the interpretation of geographical phenomena rather than a single, uniform competency ( Bendl et al., 2025). Accordingly, the observed difference may reflect the combined contribution of the coastal ecology content, contextualized learning activities, ESD-oriented tasks, and digital representations included in ESD-CoastalDTM. The findings are consistent with the rationale that geographic learning can be strengthened when students engage with geographical information and apply concepts to meaningful environmental problems. Nevertheless, because the present study compared an integrated digital intervention with conventional printed textbooks, it cannot establish that any single component was responsible for the observed difference. The reported effect size should therefore be interpreted as evidence of a difference between the instructional conditions, not as an isolated estimate of the effect of ESD pedagogy.

One possible explanation for the observed outcome is the contextualization of coastal ecology concepts within environments familiar to students. Learning activities that connect geographical concepts with recognizable environmental conditions may help students relate abstract knowledge to observable processes, including coastal erosion, changes in coastal ecosystems, and interactions between human activities and environmental systems. A second possible explanation concerns the integration of sustainability-oriented tasks, which may encourage students to examine environmental problems from multiple perspectives and consider the consequences of alternative responses. These interpretations are compatible with contemporary discussions of geographical thinking, which emphasize the importance of clearly defining the cognitive processes and competencies targeted by geography education ( Bendl et al., 2025). However, the present study did not isolate contextualization or ESD scaffolding as separate experimental factors. Consequently, these mechanisms should be treated as theoretically plausible explanations rather than demonstrated mediators of the observed outcome. Establishing their individual contributions would require a comparative design in which instructional content, learning time, and digital features are controlled while the pedagogical components are systematically varied.

The subscale results further indicate that the intervention outcomes differed in magnitude across the four dimensions of geographic literacy. Sustainability Orientation showed the largest reported effect size (d = 0.91), followed by Environmental Knowledge (d = 0.81), Spatial Thinking (d = 0.78), and Geospatial Skills (d = 0.71). The relatively large effect for Sustainability Orientation may be consistent with the intervention’s explicit emphasis on sustainability-related reflection and decision-making. Nevertheless, this result warrants particular caution because the construct was assessed through self-reported responses following a six-week intervention. Students may have developed greater awareness of sustainability issues, but their responses may also have been influenced by social desirability, perceived expectations, or increased familiarity with the environmental messages emphasized in the learning materials. These are plausible alternative explanations, not biases demonstrated by the present data. Research by Mundt et al. (2024) on retrieval practice in ESD examined retention, attitudes, and self-reported behavior as distinct outcomes, illustrating the importance of distinguishing between what students report and what can be demonstrated through other measures. Similarly, longitudinal research by Olsson et al. (2022) examined the development of secondary students’ action competence for sustainability over time, highlighting the importance of evaluating sustainability-related outcomes beyond immediate post-intervention measurements. In the present study, therefore, d = 0.91 should be interpreted as a comparatively large short-term difference in reported Sustainability Orientation, not as evidence of durable value change or established pro-environmental behavior. Follow-up measurements and behavioral indicators would be needed to assess the persistence and practical significance of this outcome.

The findings must also be interpreted in light of the quasi-experimental design. Although the observed differences favor ESD-CoastalDTM, the use of conventional printed textbooks as the comparison condition leaves several explanations unresolved, including the contribution of digital interactivity, novelty, differential student engagement, and the specific ESD-oriented pedagogy. These factors may have operated together, and the current design cannot disentangle their respective contributions. In addition, statistical adjustment for pre-test scores can account for measured baseline differences but does not eliminate all potential confounding associated with non-random assignment or differences in implementation. The results therefore support the conclusion that participation in the integrated ESD-CoastalDTM condition was associated with more favorable geographic literacy outcomes than participation in the textbook-based condition; they do not establish that ESD integration alone caused the differences. Future studies should compare ESD-integrated digital materials with an equally interactive digital condition using comparable content, instructional time, and teacher support. They should also document implementation fidelity, incorporate measures that distinguish reported sustainability orientation from observable behavior, and conduct longitudinal follow-up. Such designs would provide stronger evidence regarding the specific contribution of ESD pedagogy, the durability of sustainability-related outcomes, and the conditions under which digital coastal ecology materials can support different dimensions of geographic literacy.

### 5.3 Differential effects across geographic literacy dimensions

The differences in effect sizes across the four dimensions of geographic literacy indicate that the outcomes of ESD-CoastalDTM were not uniform. Sustainability Orientation showed the largest reported effect size (d = 0.91), followed by Environmental Knowledge (d = 0.81), Spatial Thinking (d = 0.78), and Geospatial Skills (d = 0.71). Although this pattern indicates a comparatively larger short-term difference in Sustainability Orientation, it does not establish that sustainability-related dispositions are inherently more responsive to brief educational interventions than cognitive or technical competencies. Longitudinal research by Olsson et al. (2022) on secondary students’ action competence for sustainability highlights the importance of examining how sustainability-related outcomes develop over time rather than relying exclusively on immediate post-intervention measurements. In the present study, the comparatively large effect for Sustainability Orientation may reflect increased awareness of coastal environmental issues and greater recognition of sustainability principles. However, because this dimension was assessed through self-reported responses, the observed difference may not necessarily represent stable changes in students’ values or established pro-environmental behavior. Koller et al. (2023) demonstrated that self-reported pro-environmental behavior can be sensitive to measurement-related bias, underscoring the need for caution when interpreting responses concerning environmentally desirable attitudes and behaviors. Although social desirability bias was not directly measured in the present study, it remains a plausible alternative explanation that cannot be excluded. Accordingly, the effect size of d = 0.91 should be interpreted as a comparatively large short-term difference in reported Sustainability Orientation between the experimental and control groups, rather than evidence of durable value change or actual environmental action.

In contrast, Geospatial Skills exhibited the smallest reported effect size (d = 0.71) and greater variability in the experimental group’s post-test scores (SD = 3.4) than in the control group (SD = 2.6). This pattern indicates that students’ performance in the geospatial dimension was more dispersed in the experimental condition, although the descriptive statistics alone cannot establish the reasons for this variation. Schulze (2021), in a systematic review of GIS-supported learning, highlighted the importance of considering the interaction between technological tools, learning contexts, subject content, and pedagogical approaches when examining educational outcomes. This perspective suggests that the contribution of geospatial technology depends not only on its availability but also on how it is integrated into learning activities. Within ESD-CoastalDTM, interpreting maps, annotating satellite imagery, and connecting spatial representations with coastal processes may require procedural and analytical skills that differ from those involved in recognizing environmental concepts or expressing sustainability-related attitudes. Differences in students’ prior experience with maps, digital interfaces, and spatial reasoning are possible explanations for the observed variability, but these factors were not independently measured and therefore cannot be confirmed as causes. The findings consequently identify an area for further instructional refinement rather than demonstrate that the six-week intervention was insufficient. Future implementations could incorporate sequenced geospatial tasks, worked examples, guided interpretation of maps and satellite imagery, and progressively more independent spatial analysis. Measuring students’ baseline geospatial proficiency would also help determine whether differentiated scaffolding is associated with more consistent learning outcomes.

Taken together, the findings suggest that geographic literacy should be evaluated as a multidimensional outcome rather than solely through an overall score. The comparatively larger effect for Sustainability Orientation may reflect increased awareness of sustainability issues or students’ responses to the sustainability messages emphasized in the instructional materials, whereas the comparatively smaller effect for Geospatial Skills may reflect the different demands involved in applying spatial representations to geographical problems. These interpretations remain provisional because the study did not experimentally isolate the mechanisms underlying each outcome. Furthermore, the quasi-experimental design limits causal attribution: the comparison between ESD-integrated digital materials and conventional printed textbooks cannot distinguish the contribution of ESD pedagogy from digital interactivity, instructional novelty, or other differences between the learning conditions. Therefore, the observed pattern does not establish that ESD integration alone produced the larger Sustainability Orientation effect or that geospatial skills necessarily require longer interventions. Future research should incorporate an active digital comparison condition, delayed assessments of sustainability orientation, behavioral or performance-based measures, and baseline assessments of geospatial proficiency. Such designs would provide stronger evidence regarding whether short-term differences persist, whether reported sustainability orientation corresponds to observable environmental action, and which instructional supports are most appropriate for developing each dimension of geographic literacy.

### 5.4 Limitations

Several limitations should be considered when interpreting the findings. First, the quasi-experimental design limits causal attribution because adjusting for pre-test scores using ANCOVA cannot eliminate selection bias or unmeasured confounding. Furthermore, the comparison between ESD-CoastalDTM and conventional printed textbooks does not isolate the contribution of ESD pedagogy from digital interactivity, instructional novelty, or differential student engagement. These factors remain plausible alternative explanations rather than demonstrated causes of the observed outcomes. Future studies should incorporate an active digital control condition with comparable content, instructional duration, and teacher support, alongside implementation-fidelity monitoring, to better distinguish the contributions of the instructional approach and digital modality.

Second, Geospatial Skills showed a comparatively smaller effect size (d = 0.71) and greater post-test score variability in the experimental group (SD = 3.4) than in the control group (SD = 2.6). This pattern raises the possibility that technical spatial competencies may benefit from extended practice and differentiated scaffolding. However, the present data do not establish that the six-week intervention was insufficient, because students’ baseline geospatial proficiency and familiarity with digital mapping tools were not independently assessed. Future research should compare intervention durations, measure prior geospatial competence, and examine whether progressively scaffolded tasks produce more consistent learning outcomes.

Third, the GLS-CSS measurement structure was examined through confirmatory factor analysis (CFA) using a pilot sample of 95 students. Although the reported fit indices (CFI = 0.94, RMSEA = 0.058, and SRMR = 0.062) provide preliminary evidence of model fit, they do not constitute definitive validation of the 40-item instrument. Sample-size requirements for structural equation models depend on model complexity, factor loadings, and estimation conditions rather than on a universal participant-to-item ratio ( Wolf et al., 2013). Consequently, the stability and generalizability of the proposed measurement structure remain uncertain. Independent replication with a larger sample, accompanied by examination of item-level factor loadings, factor correlations, and additional measurement properties, is needed before the instrument can be considered established across different educational contexts.

Finally, the six-week intervention and reliance on self-reported Sustainability Orientation (d = 0.91) limit conclusions about the persistence and behavioral significance of sustainability-related outcomes. The observed difference may reflect increased environmental awareness or recognition of sustainability principles rather than stable values or established pro-environmental behavior. Social desirability bias was not directly measured and therefore remains a possible, unverified alternative explanation. Delayed post-tests and behavioral or performance-based measures are needed to determine whether these differences persist and translate into environmental action. Moreover, because the participants were drawn from coastal communities in West Java, the findings may not generalize directly to other Indonesian coastal regions with different ecological, socioeconomic, and technological conditions. Replication across diverse settings, using stronger comparison designs, independently validated instruments, and longitudinal follow-up, would strengthen the evidence regarding the effectiveness, transferability, and longer-term educational value of ESD-CoastalDTM.

## 6. Conclusion

This study developed and evaluated ESD-CoastalDTM, a six-module digital teaching resource integrating coastal ecology content with Education for Sustainable Development (ESD) competencies for senior high school students in coastal communities. Expert evaluations indicated that the materials were considered suitable for classroom implementation in terms of scientific accuracy, ESD integration, media design, and contextual relevance. The quasi-experimental findings showed a substantial difference in overall geographic literacy between students using ESD-CoastalDTM and those receiving conventional printed-textbook instruction (d = 0.84). Among the four dimensions, Sustainability Orientation exhibited the largest reported effect size (d = 0.91), whereas Geospatial Skills showed a comparatively smaller effect (d = 0.71). These findings suggest that the integrated instructional approach has potential to support multiple dimensions of geographic literacy, although the respective contributions of ESD pedagogy, digital interactivity, and instructional novelty cannot be separated by the present design. Moreover, the short intervention period and reliance on self-reported Sustainability Orientation do not establish lasting changes in students’ values or pro-environmental behavior.

The study contributes a context-specific approach to developing digital teaching materials that connects coastal ecology, geographic literacy, and sustainability-oriented learning. The GLS-CSS instrument also offers a potential assessment resource, although further validation with larger and more diverse samples is needed before its measurement structure can be considered established across educational contexts. For educational practice, the findings provide a basis for further testing of locally contextualized digital materials that combine ecological knowledge, spatial reasoning, and sustainability-related decision-making. Future research should replicate the intervention across coastal regions with different ecological, socioeconomic, and technological conditions; compare ESD-integrated digital materials with equally interactive digital alternatives; and document implementation fidelity to strengthen causal interpretation. Longer interventions and delayed post-tests should be used to determine whether learning gains persist, while behavioral and performance-based measures should complement self-reported sustainability orientation. In addition, studies should investigate whether extended practice and differentiated scaffolding improve geospatial skills, particularly among students with different levels of prior spatial proficiency. Together, these steps would clarify the effectiveness, transferability, and longer-term educational value of ESD-integrated digital coastal ecology education.

## Data Availability

The data supporting the findings of this study are publicly available in the Zenodo open repository. The dataset includes anonymised GLS-CSS pre- and post-test scores, ANCOVA results, validity ratings, and instrument specifications. It does not contain any personally identifiable information. Citation: Muhammad, I. (2026). Dataset: ESD-Integrated Digital Teaching Materials for Coastal Ecology — Geographic Literacy Study (Muhammad, I et al., 2026) [Data set]. Zenodo. https://doi.org/10.5281/zenodo.20569465.
