# Supplementary material for: ESD-Integrated Digital Learning for Geographic Literacy in Coastal Secondary Schools: Development and Evaluation
Source: F1000Res. 2026 Oct 26;15:1043. Originally published 2026 Jun 30. [Version 2] doi: 10.12688/f1000research.184677.2 (PMC13755619; doi:10.12688/f1000research.184677.2)
Supplement: Peer review history

# Reviewer report: Reviewer response for version 2

**DOI:** 10.5256/f1000research.213034.r534452

**Published:** 2026-11-11

**Authors:** Soon Singh Bikar Singh

**Affiliations:** 1 Universiti Malaysia Sabah, Kota Kinabalu, Sabah, Malaysia

Overall, the article addresses a relevant and contemporary issue in geography education. However, from a geography education research perspective, the manuscript still raises important questions concerning its theoretical contribution, geographical conceptualisation, research design, instrument validity, statistical reporting and the interpretation of findings.

 b) Abstract

- The background does not sufficiently explain the specific geographical literacy problem experienced by coastal secondary students.
- The abstract reports strong effect sizes but does not provide the actual ANCOVA statistic, such as the F-value and degrees of freedom.

C. Introduction

 1. Insufficient emphasis on the actual problem in geography education.

 The introduction discusses coastal environmental challenges extensively. However, it provides limited empirical evidence about the specific weaknesses in students' geographic literacy.

 For example:

- Are students unable to interpret geographical maps?
- Do they have difficulties understanding coastal spatial processes?
- Are they unable to explain relationships between human activities and coastal degradation?
- Do they lack the ability to use geospatial information in geographical decision-making?

D. Theoretical Framework and Literature Review

 1. The theoretical framework lacks a sufficiently integrated conceptual model.

 The manuscript discusses ESD, PBE, place attachment and ADDIE, but the relationships between these frameworks remain largely descriptive.

 2. Geographical theories and perspectives are underdeveloped.

 The literature review would benefit from greater engagement with geographical thinking, spatial cognition, geographical inquiry and the concept of geographical scale.

 E. Methodology

 1. School-level confounding

 The research involves four schools and 148 students. However, the article does not adequately establish how the experimental and control conditions were distributed across the four schools.

 If two schools were assigned to the experimental condition and two to the control condition, differences in school culture, teacher experience, infrastructure and student characteristics could influence the results.

 The authors must clarify:

- How many schools participated in each condition?
- How many classes were involved?
- Were the same teachers responsible for both groups?
- How was consistency of teaching maintained?

This is important because students are nested within classes and schools. Conventional ANCOVA may underestimate standard errors if clustering is ignored.

 F. Results and Findings

 2. Statistical reporting is incomplete.

 Table 2 reports adjusted means, Cohen's d and p-values, but does not provide the ANCOVA F-statistic, degrees of freedom or confidence intervals for the principal between-group comparison.

 More importantly, the table reports separate p-values for the experimental and control groups. The authors must distinguish within-group pre-post comparisons from the actual ANCOVA between-group effect.

 A significant change in the experimental group and a non-significant change in the control group do not, by themselves, establish a statistically significant difference between groups.

 3. The effect-size calculations require verification.

 This is a particularly important technical issue.

 The reported overall post-test means are 72.4 and 52.1, with standard deviations of 8.3 and 7.4, respectively. Yet the reported Cohen's d is 0.84.

 Likewise, the subscale means and standard deviations in Table 3 do not appear to produce the reported effect sizes under the conventional pooled-standard-deviation calculation.

 This does not automatically establish that the values are incorrect because the authors may have used an adjusted or otherwise different standardisation procedure. However, the method must be explicitly explained and the calculations independently checked against the dataset.

Is the work clearly and accurately presented and does it cite the current literature?

Yes

If applicable, is the statistical analysis and its interpretation appropriate?

Yes

Are all the source data underlying the results available to ensure full reproducibility?

Yes

Is the study design appropriate and is the work technically sound?

Yes

Are the conclusions drawn adequately supported by the results?

Yes

Are sufficient details of methods and analysis provided to allow replication by others?

Partly

Reviewer Expertise:

Geography Education, Educational Psychology

I confirm that I have read this submission and believe that I have an appropriate level of expertise to confirm that it is of an acceptable scientific standard, however I have significant reservations, as outlined above.

# Reviewer report: Reviewer response for version 1

**DOI:** 10.5256/f1000research.203844.r501812

**Published:** 2026-08-06

**Authors:** Sugiyanto Sugiyanto

**Affiliations:** 1 Universitas Sebelas Maret, Surakarta City, Indonesia

**Key Review Findings & Primary Concerns:**

- **Methodological Confounding (Active Control Gap): **The control group received standard printed textbook instruction, making it impossible to separate the effect of the ESD pedagogical framework from the novelty effect of using interactive digital media (Hawthorne effect).
- **Sample Size Constraints for Factor Analysis: **The Confirmatory Factor Analysis (CFA) for instrument validation was performed on a sample of n=95, which is statistically underpowered for a 40-item scale.
- **Discrepancies in Skill Dimensions: **The Geospatial Skills subscale exhibited noticeably lower effect sizes and higher variance (SD = 3.4) compared to other dimensions, requiring deeper qualitative and empirical explanation.
- **Short-Term Measurement of Affective Constructs: **A massive shift in Sustainability Orientation (d = 0.91) over a 6-week intervention raises questions regarding construct stability vs. self-reporting bias.
  The table below outlines the itemized peer review comments, critical questions for the authors, and specific actionable revision targets structured by manuscript section.
**Manuscript Section**
**Reviewer Comments & Critical Questions**
**Actionable Revision Target / Author Requirement**
  Title & Abstract
  1. The title is overly descriptive and long.
  2. Abstract reports strong statistical effects (d=0.84, d=0.91) but omits the main methodological limitation (quasi-experimental design without active digital control).
  • Question: Can the authors sharpen the title to highlight the core innovation?
  1. Shorten the title to be more impactful (e.g., 'ESD-Integrated Digital Learning Enhances Geographic Literacy in Coastal Secondary Schools: An R&D Study').
  2. Explicitly note the quasi-experimental nature and call for longitudinal tracking in the abstract conclusion.
  1. Introduction
  1. The rationale for why ESD-integrated DTMs are superior to standard digital geography tools is not sufficiently articulated.
  2. Novelty statement at the end of Section 1 is generic.
  • Question: What specific theoretical mechanism in ESD drives spatial and environmental reasoning better than standard multimedia?
  1. Strengthen the theoretical bridge between ESD competencies (systems/anticipatory thinking) and geographic literacy dimensions.
  2. Clearly delineate the 3-fold novelty (content contextuality, ESD framework integration, and validated GLS-CSS scale).
  2. Theoretical Framework
  1. The distinction between short-term dispositional shift and long-term behavioral change in ESD is blurred.
  2. Place attachment theory is mentioned later in discussion but missing from the literature review.
  1. Incorporate Place-Based Education (PBE) and Place Attachment Theory (Kudryavtsev et al.) into Section 2.2.
  2. Frame Sustainability Orientation as an 'initial dispositional awareness' rather than permanent behavioral transformation.
  3. Methodology & Design
  1. Passive Control Group Constraint: Comparing DTM vs traditional textbook creates a modality confounding variable (Hawthorne / Novelty Effect).
  2. Instrument CFA Validation: n=95 is too small for 40 items (rule of thumb is 5-10 responses per item, i.e., n≥200).
  • Questions:
  a) How did you control for teacher enthusiasm/bias across the 4 schools?
  b) Why was an active digital control group omitted?
  1. Add an explicit 'Methodological Limitations' paragraph in Section 3.1 & 5.4 acknowledging the lack of an active digital control group.
  2. State transparently in Section 3.4 that n=95 serves as an initial pilot CFA calibration, recommending larger sample replication.
  3. Describe teacher orientation procedures to demonstrate fidelity of implementation.
  4. Results & Findings
  1. Data presentation in Tables 1-3 is clear, but subscale variance in Geospatial Skills (SD = 3.4) is not adequately explored.
  • Question: Why did students show higher variance in Geospatial Skills compared to Sustainability Orientation (SD = 2.5)?
  1. Expand Section 4.3 text to highlight the discrepancy in Geospatial Skill mastery.
  2. Provide qualitative observation context (e.g., variations in digital infrastructure, device familiarity, or spatial reasoning baseline).
  5. Discussion
  1. Claiming d=0.91 for Sustainability Orientation over 6 weeks invites skepticism regarding social desirability bias in self-reports.
  2. Overly optimistic claims about causal impacts without acknowledging quasi-experimental boundaries.
  1. Re-frame Section 5.3 discussion: acknowledge that self-reported sustainability items may reflect heightened awareness rather than entrenched action.
  2. Explicitly discuss the lower effect on Geospatial Skills (d=0.71) as evidence that technical spatial tools require longer instructional exposure.
  6. Conclusion & Ethics
  1. Ethics statement claims IRB exemption under local guidelines, but working with minors requires explicit institutional documentation.
  2. Recommendations for future research are too brief.
  1. Verify and provide exact reference numbers for institutional research permissions.
  2. Expand future research agenda: propose RCTs with active digital controls, longitudinal behavioral tracking, and expansion to other island provinces.
  Please include articles that support the claim that the current implementation of the Merdeka Curriculum in Indonesia is having an impact and aligns with your research. Additionally, compare whether the bibliometric trends regarding the curriculum in geography align with the model you have developed. You may also include references on enhancing differentiated instruction to improve the quality of learning (SDG 4).

Is the work clearly and accurately presented and does it cite the current literature?

Yes

If applicable, is the statistical analysis and its interpretation appropriate?

Partly

Are all the source data underlying the results available to ensure full reproducibility?

Yes

Is the study design appropriate and is the work technically sound?

Yes

Are the conclusions drawn adequately supported by the results?

Partly

Are sufficient details of methods and analysis provided to allow replication by others?

No

Reviewer Expertise:

I have expertise in research, particularly in geography teaching models, the evolution of geography education, and approaches to teaching geography

I confirm that I have read this submission and believe that I have an appropriate level of expertise to confirm that it is of an acceptable scientific standard, however I have significant reservations, as outlined above.

# Response

**Published:** 2026-10-14

**Authors:** Muhammad Ikhsan

**Affiliations:** Geografi Education, Universitas Khairun, Ternate, North Maluku, Indonesia

**RESPONSE TO REVIEWER**

**Manuscript title:** *Development of Digital Teaching Materials on Coastal Ecology Based on Education for Sustainable Development to Enhance Geographic Literacy Among Senior High School Students in Coastal Areas*

 Dear Reviewer 2,

 We sincerely thank you for your careful evaluation of our manuscript and for providing constructive comments on its theoretical foundation, methodological rigor, interpretation of findings, and practical implications. Your feedback has helped us identify areas requiring more precise claims, stronger connections with previous research, and greater transparency regarding the limitations of the study.

 In response, we have revised the manuscript across the Introduction, Theoretical Framework, Methodology, Results and Findings, Discussion, Limitations, Conclusion, and Ethical Considerations sections. The revisions focus on moderating claims of novelty and effectiveness, clarifying the preliminary status of the GLS-CSS psychometric evaluation, expanding the interpretation of differences across geographic literacy dimensions, and strengthening recommendations for future research.

 Our point-by-point responses are provided below.

**Comment 1: Title and Focus of the Study**

**Reviewer’s comment:** The title should be shortened and refined to improve clarity, emphasize the study’s main contribution, and better reflect its research scope.

**Response:**

 Thank you for this valuable suggestion. In response, we have revised the title to make it more concise and to communicate the study’s central focus more clearly. The revised title emphasizes the integration of Education for Sustainable Development (ESD) into digital learning, its application to geographic literacy in coastal secondary schools, and the study’s dual focus on material development and evaluation.

**Original title:** *Development of Digital Teaching Materials on Coastal Ecology Based on Education for Sustainable Development to Enhance Geographic Literacy Among Senior High School Students in Coastal Areas*

**Revised title:** *ESD-Integrated Digital Learning for Geographic Literacy in Coastal Secondary Schools: Development and Evaluation*

 This revision reduces unnecessary wording while retaining the three principal elements of the study: the ESD-integrated digital learning approach, geographic literacy as the learning outcome, and the development-and-evaluation research design. The revised title also avoids implying that the study isolates the independent effect of ESD pedagogy from the influence of digital learning media. Consistent with the quasi-experimental design, the manuscript now frames the findings as an evaluation of the integrated instructional package compared with conventional textbook-based instruction.

**Comment 2. Introduction: Research Novelty and Claims of Effectiveness**

**Reviewer’s comment:**

 The novelty claim should be more cautious. The validity of the GLS-CSS instrument requires careful qualification because the CFA was conducted with a sample of 95 students. Furthermore, the Introduction should not imply that the independent effect of ESD has been established when the intervention was compared with conventional textbook instruction.

**Response:**

 Thank you for this important observation. We agree that the novelty and effectiveness claims should be aligned with the scope of the study and its methodological design.

 We have refined the Introduction to position ESD-CoastalDTM as a context-specific contribution to digital geography education rather than claiming that it represents a definitive or unprecedented pedagogical innovation. We have also moderated statements concerning the GLS-CSS instrument by distinguishing its initial psychometric evaluation from comprehensive validation across educational contexts.

 In addition, we have clarified that the study evaluates an integrated package of ESD-oriented digital teaching materials relative to conventional printed-textbook instruction. Because the comparison condition did not include an equivalent digital learning environment, the independent contribution of ESD pedagogy cannot be separated from digital modality, instructional novelty, or other differences between the conditions.

**Revisions made:**

- Moderated claims concerning the novelty and broader applicability of ESD-CoastalDTM.
- Clarified the preliminary status of the GLS-CSS psychometric evaluation.
- Aligned claims about instructional effectiveness with the quasi-experimental design.

**Comment 3. Theoretical Framework: Place-Based Education and Sustainability Orientation**

**Reviewer’s comment:**

 The distinction between short-term dispositional changes and long-term behavioural transformation is insufficiently developed. In addition, place attachment theory is discussed later in the manuscript but is not adequately established in the theoretical framework.

**Response:**

 We appreciate this recommendation. We have strengthened the theoretical rationale by connecting place-based learning with the contextualization of coastal ecology content. The revised framework explains how learning activities grounded in students’ local coastal environments may help connect geographical concepts with observable ecological processes, community experiences, and environmental challenges.

 We have also clarified the distinction between sustainability awareness, reported sustainability orientation, and sustained pro-environmental behaviour. In the revised manuscript, Sustainability Orientation is interpreted as a short-term self-reported disposition rather than evidence of permanent value transformation or established environmental action.

 This distinction provides a more appropriate conceptual basis for interpreting the comparatively large effect size observed for Sustainability Orientation while recognizing that the persistence of such changes requires longitudinal investigation.

**Revisions made:**

- Strengthened Section 2.2 by incorporating Place-Based Education and place attachment as theoretical perspectives, supported by relevant literature.
- Clarified the conceptual distinction between reported sustainability orientation and sustained environmental behaviour.
- Aligned the theoretical framework with the interpretation of the Sustainability Orientation findings in the Discussion.

**Comment 4. Methodology and Design: Active Control, CFA Sample, and Teacher Implementation**

**Reviewer’s comment:**

 The comparison between digital teaching materials and conventional textbooks introduces potential confounding related to digital modality and novelty effects. The CFA sample of 95 students is limited for a 40-item instrument. The manuscript should also clarify how teacher enthusiasm or bias was addressed across the four schools and why an active digital control condition was not included.

**Response:**

 Thank you for identifying these important methodological issues. We acknowledge that the absence of an active digital control condition limits our ability to isolate the contribution of ESD pedagogy from digital interactivity, instructional novelty, and differential student engagement. We have therefore revised the manuscript to state explicitly that the study evaluates the integrated ESD-CoastalDTM intervention relative to conventional textbook-based instruction rather than estimating the independent causal effect of ESD integration.

 Regarding the GLS-CSS instrument, we have clarified that the CFA conducted with 95 students represents an initial psychometric evaluation. The reported fit indices (CFI = 0.94, RMSEA = 0.058, and SRMR = 0.062) are presented as preliminary evidence of model fit rather than definitive validation. We have also strengthened the recommendation for independent replication with a larger sample and further assessment of item-level factor loadings, factor correlations, and measurement properties.

 We recognize that differences in teacher characteristics, classroom conditions, and implementation may also have influenced the outcomes. The manuscript therefore acknowledges the importance of documenting teacher preparation, instructional consistency, and implementation fidelity. Any description of teacher orientation or monitoring procedures will be limited to procedures that were actually conducted and can be documented.

 The absence of an active digital control condition is also acknowledged as a design limitation. Future research should compare ESD-integrated digital materials with an equally interactive digital condition that uses comparable content, instructional time, and teacher support.

**Revisions made:**

- Section 3.1: Clarified the quasi-experimental design and the limitation associated with the passive comparison condition.
- Section 3.4: Clarified the preliminary role of the CFA pilot sample.
- Section 5.4: Expanded the discussion of potential confounding, teacher-related influences, and implementation fidelity.
- Section 6: Added recommendations for active digital comparison conditions and independent psychometric validation.

**Comment 5. Results and Findings: Variability in Geospatial Skills**

**Reviewer’s comment:**

 The higher score variability in Geospatial Skills (SD = 3.4), compared with Sustainability Orientation (SD = 2.5), requires a more detailed explanation. The manuscript should consider contextual factors such as digital infrastructure, familiarity with devices, and baseline spatial reasoning.

**Response:**

 We appreciate this observation and have expanded the interpretation of the Geospatial Skills results. The revised manuscript reports that Geospatial Skills showed a comparatively smaller effect size ( *d* = 0.71) and greater post-test score variability in the experimental group (SD = 3.4) than in the control group (SD = 2.6).

 We discuss several possible explanations, including differences in prior spatial reasoning, familiarity with digital mapping tools, and opportunities to practise interpreting maps and satellite imagery. These explanations are presented as hypotheses rather than established causes because the relevant factors were not independently measured in the reported analyses.

 We have also clarified that the higher standard deviation indicates greater dispersion in students’ scores, not direct evidence of technical difficulty or inadequate instructional duration. The revised discussion therefore treats the findings as an indication that geospatial learning may benefit from further investigation of differentiated scaffolding and opportunities for guided practice.

 We agree that qualitative observations would provide useful contextual evidence. However, we will only report observations, interviews, or infrastructure-related findings if these data were actually collected and can be substantiated. We do not introduce qualitative explanations as empirical findings when they were not measured.

**Revisions made:**

- Expanded the interpretation of Geospatial Skills in Section 4.3.
- Discussed plausible explanations for score variability while distinguishing hypotheses from measured findings.
- Added recommendations for assessing baseline geospatial proficiency and testing differentiated scaffolding in future research.

**Comment 6. Discussion: Sustainability Orientation and Causal Interpretation**

**Reviewer’s comment:**

 The effect size for Sustainability Orientation ( *d* = 0.91) over six weeks raises concerns about social desirability bias in self-reported responses. The manuscript should also avoid overly optimistic causal claims and discuss the comparatively smaller effect for Geospatial Skills.

**Response:**

 Thank you for highlighting these interpretive concerns. We have revised the Discussion to distinguish short-term self-reported sustainability orientation from stable environmental values and established pro-environmental behaviour.

 The revised manuscript recognizes that the comparatively large effect size for Sustainability Orientation may reflect increased awareness of coastal environmental issues or greater recognition of sustainability principles. Potential social desirability bias is acknowledged as a plausible alternative explanation, but we do not claim that such bias occurred because it was not directly measured.

 We have also expanded the discussion of Geospatial Skills, emphasizing that the comparatively smaller effect size ( *d* = 0.71) may reflect different learning demands associated with spatial representations and technical geospatial tasks. Nevertheless, we do not conclude that the six-week intervention was definitively insufficient, because intervention duration was not experimentally varied.

 Finally, we have moderated causal language throughout the Discussion. The results are interpreted as differences associated with the integrated instructional conditions rather than proof that ESD pedagogy alone caused the observed outcomes.

**Revisions made:**

- Section 5.2: Clarified the interpretation of overall geographic literacy outcomes.
- Section 5.3: Distinguished short-term reported Sustainability Orientation from durable behavioural change and expanded the interpretation of Geospatial Skills.
- Section 5.4: Added limitations related to self-reporting, possible social desirability bias, and the quasi-experimental design.

**Comment 7. Conclusion and Ethical Considerations**

**Reviewer’s comment:**

 The ethical statement claims an IRB exemption based on local guidelines, although research involving minors requires explicit institutional documentation. The recommendations for future research are also too brief.

**Response:**

 We appreciate this important comment concerning ethical transparency. We have revised the approach to the Ethical Considerations section to distinguish administrative research permissions from formal ethics approval or an institutional determination of exemption.

 The administrative permission reference previously reported in the manuscript (No. 421/235/SMAN.4-TTE/2026) is identified as a school-related research permission and will not be represented as an ethics approval or exemption reference unless the original documentation explicitly establishes that status. The final statement must reflect the actual institutional determination, including the authorized committee or office and the relevant reference number and date, where applicable. Parental or legal-guardian consent and student assent will also be described according to the procedures actually followed and documented.

 We have also expanded the recommendations for future research. These include randomized controlled trials, where feasible, with active digital control conditions; longitudinal tracking of sustainability-related outcomes using behavioural or performance-based measures; replication across coastal provinces with different ecological and socioeconomic characteristics; independent validation of GLS-CSS; and investigation of differentiated geospatial scaffolding.

 Regarding the Merdeka Curriculum, geography education, and SDG 4, we have strengthened the contextual discussion while distinguishing conceptual alignment and bibliometric trends from direct evidence of causal effectiveness. The manuscript does not claim that the Merdeka Curriculum independently caused the observed learning outcomes.

**Revisions made:**

- Revised the Ethical Considerations statement to require accurate reporting of documented institutional ethics status.
- Expanded Section 5.4, *Limitations*, and Section 6, *Conclusion*.
- Strengthened the discussion of the educational context, relevant geography education research, and differentiated instruction.

**Comment 8. Recommendations for Future Research**

**Reviewer’s comment:**

 The recommendations for future research require greater detail and specificity.

**Response:**

 Thank you for this suggestion. We have expanded the future research agenda to address the methodological, measurement, and contextual limitations identified in the review.

 First, randomized controlled trials, where feasible, should incorporate active digital comparison conditions to help distinguish ESD pedagogy from digital modality and instructional novelty. Second, longitudinal studies should use delayed post-tests and behavioural or performance-based indicators to determine whether short-term changes in Sustainability Orientation persist and translate into observable environmental action. Third, replication across coastal provinces in Indonesia, including regions with different ecological, socioeconomic, and technological conditions, would help assess the transferability of the materials.

 In addition, further psychometric research should examine the GLS-CSS measurement structure using larger independent samples. Future studies should also compare different intervention durations and levels of geospatial scaffolding to determine whether extended practice and differentiated support improve learning outcomes among students with different levels of prior spatial proficiency.

**Revisions made:**

- Expanded Section 5.4, *Limitations*.
- Strengthened Section 6, *Conclusion*, with specific methodological and contextual recommendations.

We sincerely thank Reviewer 2 for the constructive comments and recommendations. The review has helped us improve the transparency of the methodological reporting, refine the interpretation of the results, strengthen the theoretical discussion, and identify more specific directions for future research.

 We believe that the revised manuscript presents a more balanced account of the contribution and limitations of ESD-CoastalDTM. We hope that the revisions adequately address the reviewer’s concerns and that the manuscript will be suitable for further consideration.

 Sincerely,

**Muhammad Ikhsan**

 Corresponding Author

 On behalf of all co-authors

# Reviewer report: Reviewer response for version 1

**DOI:** 10.5256/f1000research.203844.r501807

**Published:** 2026-07-29

**Authors:** Bayu Wijayanto

**Affiliations:** 1 Padang State University, Padang, Indonesia

This manuscript addresses a timely and relevant issue by integrating Education for Sustainable Development (ESD) principles into digital teaching materials for coastal ecology. The study is particularly valuable because it focuses on students in coastal areas, where contextualized geographic and environmental education is highly important. The research objective is clearly stated, and the use of the ADDIE model provides a systematic framework for the development and evaluation of the digital teaching materials. The methodological approach is also well structured, involving expert validation and a quasi-experimental design to examine effectiveness. The reported results demonstrate strong validity and a meaningful improvement in students' geographic literacy, as reflected by the substantial effect sizes. The emphasis on sustainability orientation as the strongest outcome further highlights the potential contribution of the intervention to ESD. Overall, the study offers a relevant and empirically supported contribution to geography and sustainability education, particularly in coastal and archipelagic contexts. The manuscript has good potential to contribute to the development of innovative, contextualized, and sustainability-oriented digital learning resources.

Is the work clearly and accurately presented and does it cite the current literature?

Yes

If applicable, is the statistical analysis and its interpretation appropriate?

Yes

Are all the source data underlying the results available to ensure full reproducibility?

Yes

Is the study design appropriate and is the work technically sound?

Yes

Are the conclusions drawn adequately supported by the results?

Yes

Are sufficient details of methods and analysis provided to allow replication by others?

Yes

Reviewer Expertise:

geography education

I confirm that I have read this submission and believe that I have an appropriate level of expertise to confirm that it is of an acceptable scientific standard.
